# Improving the performance of perovskite solar cells by extending π-conjugation system†

**DOI:** 10.1039/d4ra03173c

**Published:** 2024-06-18

**Authors:** Babak Pashaei

**Affiliations:** a Department of Inorganic Chemistry, Faculty of Chemistry, University of Mazandaran Babolsar Iran B.pashaei@umz.ac.ir

## Abstract

In perovskite solar cells (PSCs), hole transporting materials (HTMs) play a critical role in determining the stability and efficiency of the devices. However, the high cost and complex synthesis processes associated with conventional HTMs can hinder their widespread applications. This work presents a low-cost and efficient HTM, namely *N*,*N*′-(naphthalene-1,5-diyl)bis(1-(dibenzo[*a*,*c*]phenazin-11-yl)-1-phenylmethanimine) (PEDN), based on a naphthalene core with an extended π-conjugation system for improving the performance of PSCs. The PEDN was synthesized *via* a facile two-step condensation method, eliminating the need for expensive catalysts such as BINAP. The newly developed HTM with an extended π-conjugation length was compared with BEDN and spiro-OMeTAD as the benchmark HTM, in terms of their optical, electrochemical, hole mobility properties, and efficiency in PSCs. The PEDN showed suitable highest occupied molecular orbital levels (HOMOs), good hole mobilities, as well as strong hydrophobicities. The extended π-conjugation system in PEDN contributes to the stability of the solar cells. The PSCs fabricated with PEDN achieved a high efficiency of 18.61%, comparable to the efficiency obtained using the commonly used HTM spiro-OMeTAD (19.68%). Furthermore, the cost-effectiveness of PEDN makes it a suitable alternative to spiro-OMeTAD for PSC applications.

## Abstract 2

In this study, a novel naphthalene-based hole-transporting material was synthesised and studied for their potential application in perovskite solar cells.

## Graphical abstract

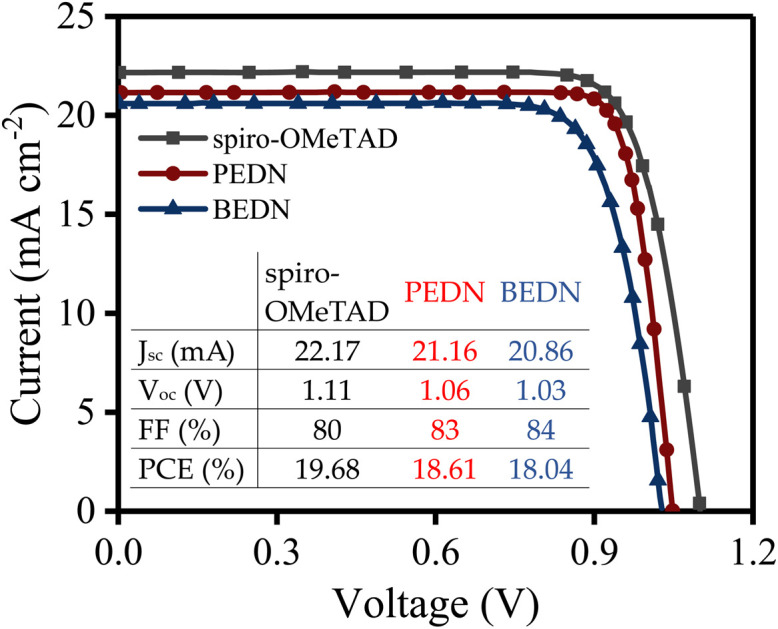

## 1 Introduction

At present, the world's energy requirements are fulfilled *via* various energy sources, with fossil fuels being the primary source.^1^ In light of the finite nature of fossil fuel resources and the urgent need to mitigate anthropogenic climate change, the transition to a diversified and sustainable energy system centered on renewable sources is of paramount importance.^2^ Solar energy, characterized by its abundance and minimal environmental impact, is a prominent renewable energy source. Photovoltaic devices, serving as the primary means of converting incident photon energy into electrical current, are commonly referred to as conventional solar cells. These devices utilize semiconductor materials and fundamental physical processes to facilitate this energy conversion.^3–5^

About 16 years have passed since perovskites, known as third-generation solar cells, were used as an organic–inorganic hybrid material for light absorption.^6^ Perovskite materials, renowned for their optoelectronic properties, are gaining significant attention as cost-effective light absorbers in photovoltaic devices. Their exceptional characteristics, including an optimal and direct bandgap, coupled with a high absorption coefficient, make them promising candidates for efficient solar energy conversion.^7^ The overall structure of perovskite is usually ABX_3_, where A, B, and X are cation, cation and anion, respectively (halides group). The cation A is larger than the cation B.^8^ The most common perovskite material has the chemical formula CH_3_NH_3_PbX_3_. In this type of perovskite, the electron–hole diffusion length is approximately one micron, while its energy bandgap is around 1.5 eV. The rapid increase in power conversion efficiency (PCE) of perovskite solar cells (PSCs) in a short time period is noteworthy; it has surpassed 26% at the laboratory scale.^9^ The PSCs are composed of several layers, including counter electrodes, hole transporting layer (HTL), light-absorbing perovskite layer, electron transporting layer (ETL), and a transparent conductive oxide (TCO)-coated glass substrate.^10^ The ETL plays a crucial role in transporting electrons from the perovskite layer to the cathode or anode and reducing electron recombination by optimizing the interface between the electrode and the light-absorbing layer. However, PSCs face challenges such as low stability and flexibility, which have hindered their commercialization.^11^

The HTMs play a vital role in perovskite solar cells by efficiently extracting holes from the perovskite layer and facilitating their rapid transfer to the back electrode.^12–14^ Additionally, HTMs serve as a protective barrier between the perovskite and the metal electrode, preventing degradation and moisture ingress. Ideal HTMs should possess several key properties, including suitable energy level alignment, high hole mobility, good solubility, excellent thermal and photochemical stability, high hydrophobicity, and cost-effectiveness.^15^ The commonly used HTMs in PSCs, such as spiro-OMeTAD and PTAA, are relatively expensive to synthesize.^16^ Therefore, the development of low-cost HTMs with high photovoltaic performance is crucial for the commercialization of PSCs. In addition, the mobility of holes in the HTMs can have a significant effect on improving the efficiency of solar cells. One effective strategy to improve the hole mobility in HTMs is to introduce electron-deficient groups to create donor–acceptor structures.^17,18^ These donor–acceptor structures promote intermolecular interactions and intramolecular charge delocalization, leading to enhanced charge transport.^19^ Additionally, by carefully selecting the donor and acceptor units, the energy levels of the frontier orbitals can be fine-tuned to optimize the energy level alignment with the perovskite layer.

Through the strategic extension of the conjugated π-system within BEDN,^20^ it has successfully enhanced the stability and performance of photovoltaic devices. Herein, a novel HTM named *N*,*N*′-(naphthalene-1,5-diyl)bis(1-(dibenzo[*a*,*c*]phenazin-11-yl)-1-phenylmethanimine) (PEDN) has been synthesized and investigated as an efficient and stable HTM for PSCs. The newly synthesized HTM exhibited superior hole mobility exceeding 10^−4^ cm^2^ V^−1^ s^−1^, thermal stability up to 141 °C, and an appropriate alignment of its HOMO energy level with that of the perovskite. The photovoltaic device incorporating PEDN showed superior performance compared to the device employing BEDN, achieving a PCE of 18.61%. Notably, the production cost of 1 g of PEDN is significantly lower than that of previously reported HTMs, rendering it an economically viable option for large-scale applications.

## 2 Experimental

### 2.1 Synthesis of PEDN

**Fig. 1:**
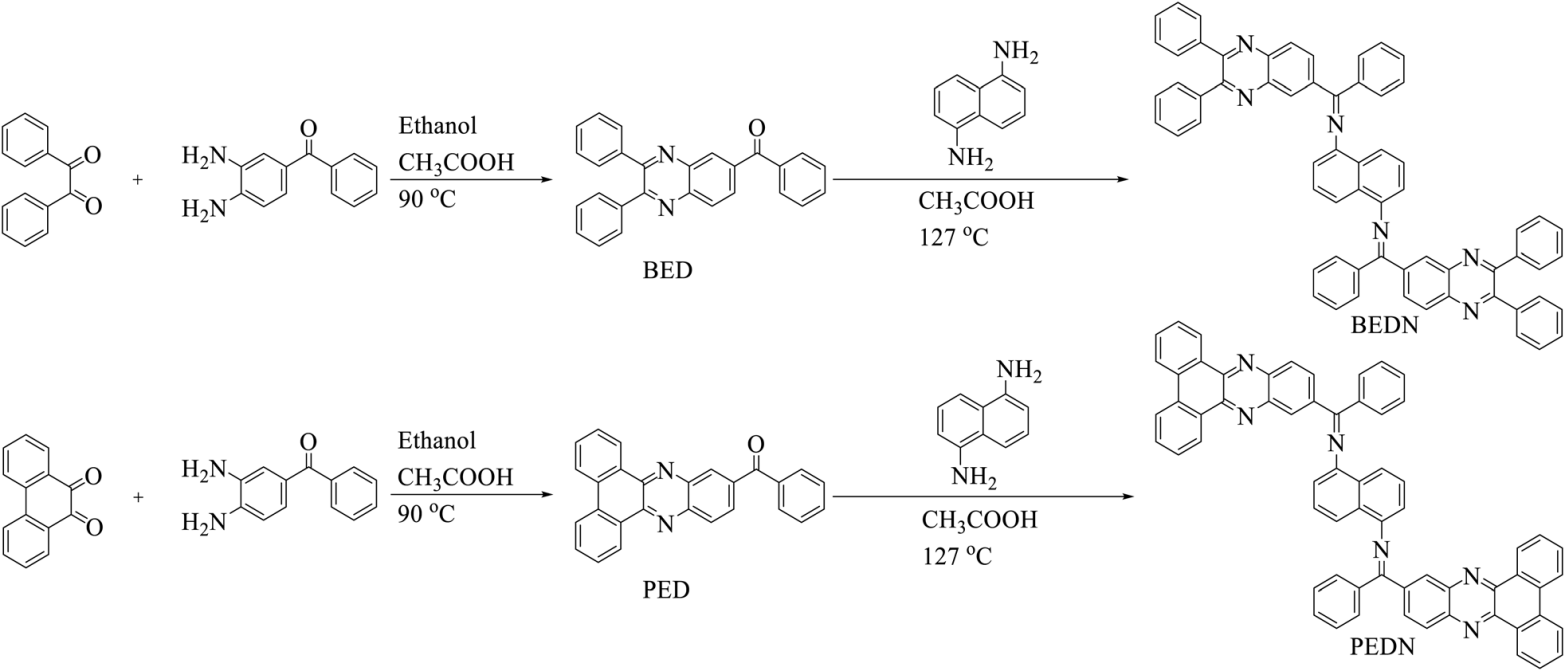
The synthesis route of PEDN and BEDN HTMs.

The route of PEDN synthesis is schematically shown in Fig. 1.^20^ Briefly, the PED was obtained from the condensation of 3,4-diaminobenzophenone (1 mmol, 212.25 mg) with phenanthrene-9,10-dione (1 mmol, 208.22 mg), which were added to a mixture of acetic acid and ethanol. After 4 h of reflux, cold ethanol was used to wash the yellow precipitate and recrystallized in ethanol. Then, the reaction between this ligand (1 mmol, 384.44 mg) and diamine naphthalene (0.5 mmol, 78 mg) under reflux for 12 h in acetic acid gave the desired product (PEDN). Acetic acid was used for recrystallization to obtain the pure product (250 mg, 56%). Analysis data: ^1^H NMR (250 MHz, DMSO), 8.33–8.27 (m, 2H), 8.18 (s, 1H), 8.84 (d, 3H), 7.71 (d, 1H), 7.64–7.58 (m, 3H), 7.46 (m, 4H), 7.37 (m, 4H). ^13^C NMR (250 MHz, DMSO), 194.20, 155.12, 153.89, 142.29, 140.08, 138.60, 137.64, 137.71, 132.36, 132.14, 131.13, 130.92, 130.28, 129.85, 130.41, 129.62, 129.16. CHN: Anal. calcd. For C_64_H_38_N_6_ (%): C, 86.27; H, 4.30; N, 9.43 found (%): C, 86.26; H, 4.31; N, 9.42 ESI-MS: *m*/*z* 890.28, [M–H]^+^.

## 3 Results and discussion

Fig. 1 shows the synthesis pathway for PEDN HTM, which was prepared following a previously reported procedure.^20^ Briefly, PEDN was synthesized *via* sequential condensation reactions between 9,10-phenanthrenequinone, 3,4-diaminobenzophenone, and naphthalene in a mixture of acetic acid (CH_3_COOH) and ethanol. The synthesized product was characterized using NMR, ESI-MS, CHN, and FT-IR (please see ESI†). The FT-IR spectrum (Fig. S1†) of the 9,10-phenanthrenequinone shows that the frequency of the stretching vibration of C=O appeared at 1677 cm^−1^. A similar absorption band corresponding to the C=O bond is also observed in the FT-IR spectrum of PED. The absence of the stretching frequency of this bond in the PEDN indicates a successful condensation of the ligands. The presence of C=N bond stretching frequencies, however, would confirm the proposed structure. Moreover, the ^1^H NMR spectrum of PEDN shows resonance peaks between 7 and 9 ppm, which is attributed to the peaks of aromatic cycles (Fig. S2†). These observations provide evidence supporting the proposed structure of PEDN.

**Fig. 2:**
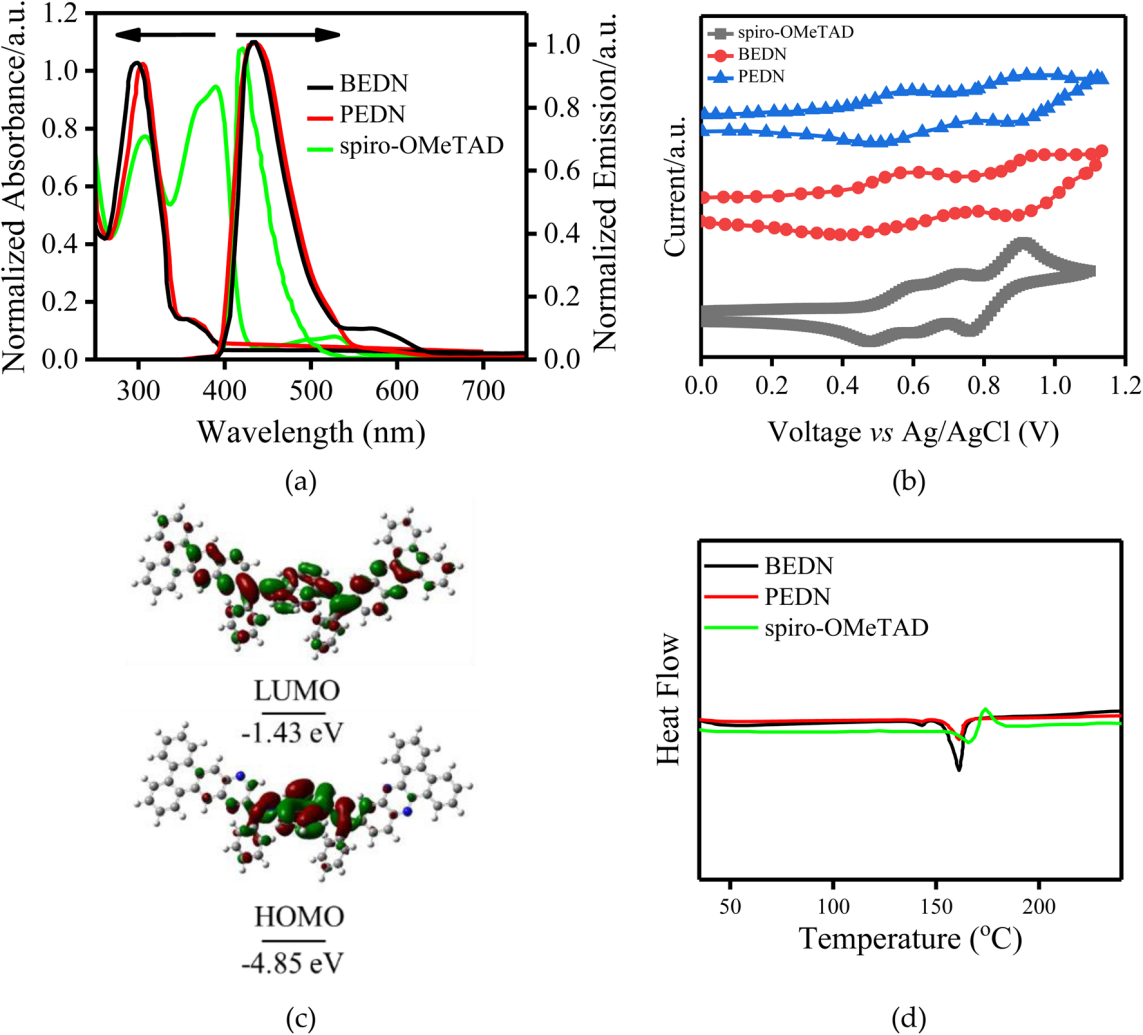
(a) UV/vis and PL spectra and (b) cyclic voltammograms of the PEDN, BEDN, and spiro-OMeTAD HTMs. (c) HOMO–LUMO energy levels and molecular distributions of the PEDN, BEDN, and spiro-OMeTAD HTMs from the optimized geometrical structures. (d) DSC of the PEDN, BEDN, and spiro-OMeTAD HTMs.

The optical properties of PEDN were investigated using ultraviolet-visible (UV-vis) spectroscopy. The UV-vis absorption spectrum of PEDN (shown in Fig. 2a) was compared to those of BEDN and the spiro-OMeTAD as the benchmark HTM. Both PEDN and BEDN HTMs exhibit strong absorption spectra at 301 and 307 nm, respectively. These absorption peaks are ascribed to the π–π* transitions of benzene rings within the HTM structures.^21,22^ Notably, the absorption spectrum of PEDN is red-shifted by approximately 5 nm relative to BEDN, due to presence of extended π-conjugation system and stronger intermolecular interactions.^23,24^ The maximum emission spectra for BEDN and PEDN are observed at 427 and 436 nm, respectively. The relatively high Stokes shift of these HTMs (above 100 nm) indicates that the molecules undergo significant structural changes during excitation.^25^ Using the intersection of absorption and photoluminescence (PL) spectra, the band gap (*E*_o–o_) of the new HTM was calculated and summarized in Table 1. The calculated BG is 3.10 eV, which is approximately the same as that of BEDN HTM.

**Table 1:** Optical and electrochemical of HTMs

HTM	*λ*_abs_ a (nm)	*λ*_em_ b (nm)	*E*_o–o_ (eV)	*E*_OX_ (V)	*E*_HOMO_ (eV)	*E*_LUMO_ (eV)	*T*_g_ (°C)	*η*_quenching_	Hole mobility^c^ (cm^2^ V^−1^ s^−1^)
BEDN	301	427	3.14	0.58	−5.18	−2.02	136	0.92	9.10 × 10^−5^
PEDN	307	436	3.10	0.56	−5.16	−2.06	141	0.95	1.81 × 10^−4^
Spiro-OMeTAD	304	421	3.04	0.60	−5.21	−2.17	125	0.88	4.10 × 10^−5^

a 1 × 10^−5^ M CHCl_3_ solution.

b 1 × 10^−5^ M CHCl_3_ solution.

c Pure HTM without doping.

The energy levels of frontier orbitals of PEDN HTM were estimated from the ground state oxidation potential using cyclic voltammograms (CV).^26^ The CV curves of investigated HTMs are shown in Fig. 2b, while the corresponding electrochemical data are summarized in Table 1. The onset potential oxidation of PEDN is observed at 0.56 V, while that of BEDN occurs at 0.58 V. The HOMO energy levels of HTMs were determined by the CV, using the onset potentials and equation *E*_HOMO_ = −(*E*_ox,onset_ + 4.8) eV. The LUMO is the lowest unoccupied molecular orbital and LUMO energy levels are achieved by applying the equation *E*_LUMO_ = −(−*E*_HOMO_ − *E*_o–o_) eV. The HOMO energy level for PEDN was calculated to be −5.16 V, which suggests a suitable alignment with the perovskite HOMO energy level of −5.43 eV.^27^ To gain further insights into the energy levels of PEDN HTM, density functional theory (DFT) calculations were performed. The results shows that the HOMO energy level of PEDN is in good agreement with the perovskite HOMO energy level, providing further support for its suitability as a HTM in PSCs.^28^

Thermal stability is a critical feature for HTMs used in solar cells, as they must withstand the high temperatures encountered during device fabrication and operation. Differential scanning calorimetry (DSC) is a technique used to evaluate the thermal stability of materials by measuring the heat flow into or out of a sample as it is heated or cooled. As depicted in in Fig. 2d, the PEDN displays an elevated glass transition temperature (*T*_g_) of 141 °C, surpassing that of BEDN (136 °C) and spiro-OMeTAD (125 °C).^29^ The elevated *T*_g_ is attributed to the presence of 9,10-phenanthrenequinone moieties, which contribute to enhanced morphological stability.^30^ Furthermore, the high thermal degradation temperature of PEDN satisfies the critical requirement of photovoltaic solar cells for stability and tolerance under elevated temperatures.

**Fig. 3:**
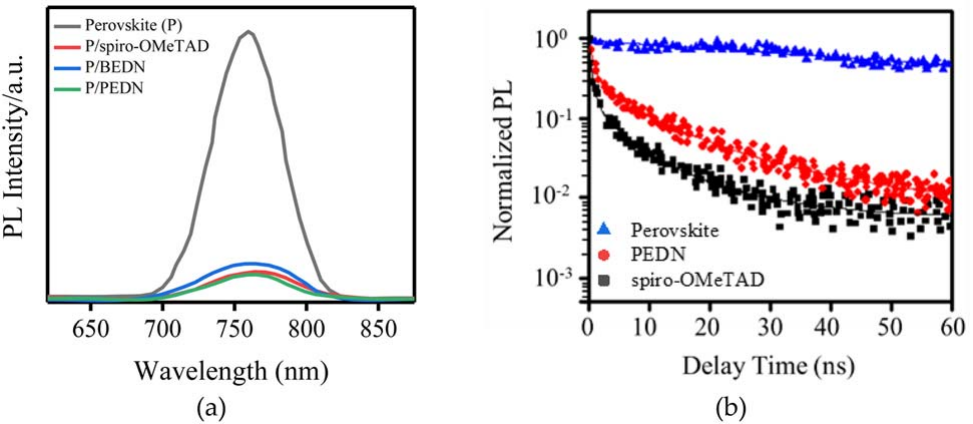
(a) The SSPL spectra for perovskite and PEDN and spiro-OMeTAD deposited onto the perovskite, photoexcited at 405 nm. (b) TRPL spectra of perovskite and BEDN, PEDN, and spiro-OMeTAD deposited onto the perovskite.

To investigate the charge transfer phenomena between perovskite and HTM, time-resolved and steady-state photoluminescence (TRPL and SSPL, respectively) were were performed on FTO/TiO_2_/perovskite and FTO/TiO_2_/perovskite/HTM samples. The SSPL spectra of all samples demonstrate a peak at 760 nm, which is attributed to the perovskite emission. The PL peak is significantly quenched in the samples with the presence of HTMs (Fig. 3a), indicating efficient charge transfer from the perovskite to the HTMs. The perovskite sample with PEDN indicates a higher *η*_quenching_ compared to spiro-OMeTAD and BEDN, suggesting improved charge extraction at the perovskite/PEDN interface. Moreover, the TRPL of PEDN deposited on the perovskite indicates the second life-time (*τ*_2_) of approximately 9.5 ns (Fig. 3b). The curves in Fig. 3b are the bi-exponential decay functions used to fit the lifetime of the perovskite/HTM.^31,32^ The *τ*_2_ may be attributed to hole extraction from the perovskite into the HTL.

To investigate and evaluate the hole mobilities of PEDB, BEDN, and spiro-OMeTAD, the devices with ITO/PEDOT:PSS/HTM/Au structure were made, where ITO and PEDOT:PSS are indium tin oxide and poly(3,4-ethylenedioxythiophene):poly(styrenesulfonate), respectively. Based on the space-charge-limited-current (SCLC) method, as shown in Fig. S3,† the hole mobility of PEDN obtained is 1.81 × 10^−4^ cm^2^ V^−1^ s^−1^, which is higher than that of BEDN and spiro-OMeTAD.

**Fig. 4:**
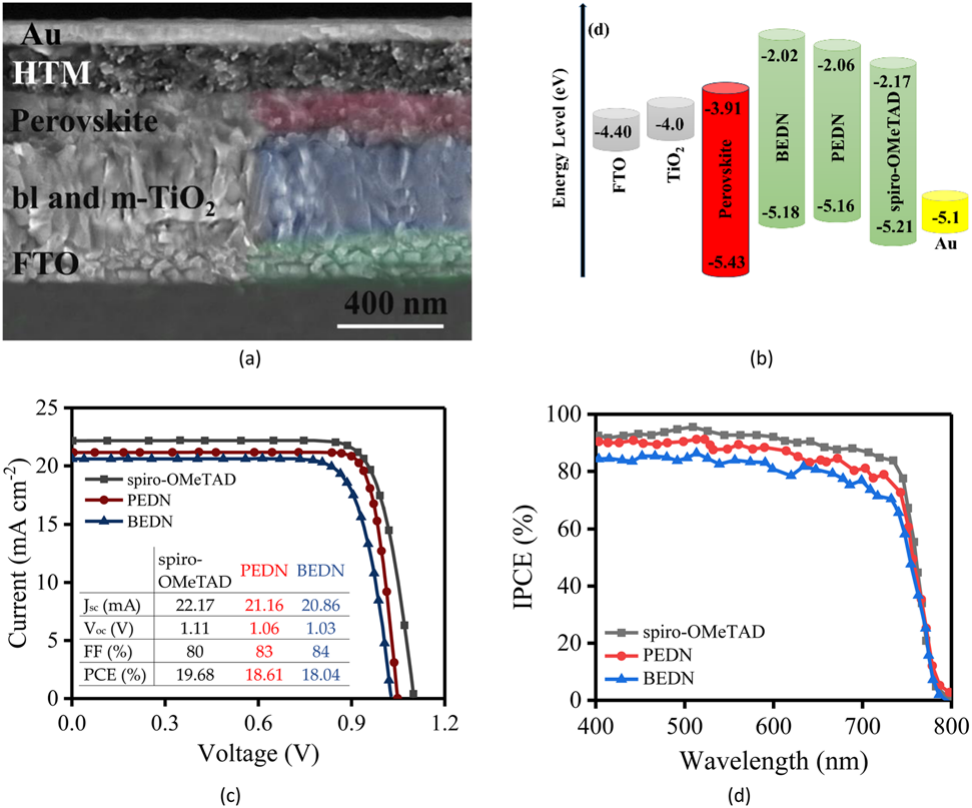
(a) Diagram of the device structure, SEM cross section of device based on PEDN. (b)Energy level diagram for each layer in PSCs. (c) *J*–*V* curves of PSCs and (d) the IPCE plots of PSCs based on PEDN, BEDN, spiro-OMeTAD HTMs.

To investigate the photovoltaic parameters, PEDN was applied as an HTM to fabricate n-i-p type PSCs with a configuration of FTO/TiO_2_/MAPbI_3_/PEDN/Au. The X-ray diffraction (XRD) analysis was performed to confirm the formation of the MAPbI_3_ perovskite thin film (Fig. S4†). As can be seen, the peaks appearing at 2*θ* of 14.08° (110), 28.5° (220), and 32° (310) correspond to the principle peaks of MAPbI_3_ perovskite, indicating the complete formation and pure tetragonal phase of the MAPbI_3_ perovskite.^33,34^ The device architecture of the standard PSC and the cross-sectional image of the cell using a scanning electron microscope (SEM) are presented in Fig. 4a. As observed in the SEM image, an optimal layer of PEDN with a thickness of approximately 250 nm uniformly covers the surface of the perovskite layer, which has a thickness of approximately 350 nm. Additionally, the gold contact can be seen on top of the HTM layer. Furthermore, Fig. S5,† which displays the corresponding cross-sectional elemental mapping of indium (In), titanium (Ti), lead (Pb), and iodine (I), confirms the uniform deposition of the perovskite films over the mesoporous mp-TiO_2_ layer. The distribution of Pb and I is relatively homogeneous throughout the perovskite film, with good penetration into the TiO_2_ layer. Fig. 4b illustrates the energy level diagram of each layer in PSCs. Based on this figure, all three HTMs exhibit nearly identical HOMO energy levels. However, the HOMO energy level of the new HTM is aligned with the energy level of the perovskite, which facilitates efficient hole extraction from the perovskite layer. Fig. 4c shows the current–voltage (*J*–*V*) curves of PEDN, BEDN, and spiro-OMeTAD-based PSCs under standard irradiation conditions (AM 1.5G, 100 mW cm^−2^) in ambient air at ≈25 °C. Photovoltaic parameters extracted from the *J*–*V* curves are summarized in the table within Fig. 4c. The PSCs fabricated with PEDN exhibit the highest PCE of 18.61%. The inferior performance of the PSCs employing BEDN compared to PEDN can be attributed to its lower *V*_OC_ due to higher HOMO level of BEDN. The *V*_OC_ of the PSCs based on PEDN and BEDN is 1.06 and 1.03 V, respectively, which are about 5 and 9 mV lower than the reference HTM, *i.e.*, spiro-OMeTAD. This observation corroborates the trend that the *J*_SC_ increases linearly with the reduction of the HTM oxidation potential.^35^ The PEDN possesses a relatively low oxidation potential compared to the BEDN, resulting in a higher *J*_SC_, as anticipated. Fig. 4d compares the incident photon-to-current efficiency (IPCE) spectra of devices based on PEDN, BEDN, and spiro-OMeTAD. The PEDN-based device exhibits a higher IPCE value than the BEDN-based device across the entire wavelength range.

The resistance of the series of PSC layers was investigated using electrochemical impedance spectroscopy (EIS).^36,37^ The Nyquist plot of the perovskite devices is shown in Fig. S6,† which is obtained in the working conditions under 1 sun illumination and *V*_OC_. In general, the charge transfer process between the perovskite layer and the back contact occurs at high frequencies, and the recombination process between the meso-TiO_2_ and perovskite layers occurs at low frequencies.^37,38^ In the equivalent circuit (inset of Fig. S6†), *R*_s_, *R*_ct_, and *C* show the series resistance due to FTO and external contact, the charge transfer resistance between perovskite/HTM interface, and capacitance. The *R*_s_ in the Nyquist diagram is the value of the starting point.^38^ The results obtained from ESI† show that charge separation at the interface of PEDN and perovskite leads to improvement in the photocurrent, FF, and PCE. The value of *R*_ct_ shows that the perovskite and HTM layers in the solar cell are uniformly formed, facilitating efficient hole transfer and contributing to the improved FF.

To study the surface morphology and charge extraction capabilities, FE-SEM and atomic force microscopy (AFM) measurements of the above perovskite films were applied (Fig. S7†). The FE-SEM image (Fig. S7a†) for the device based on PEDN displays an entire coverage film without any distinguishable pinholes or defects. In addition, the AFM-based surface topographical image of PEDN coated on the perovskite layer is shown in Fig. S7b.† The results showed that the root mean square (RMS) roughness value is 19.3 nm, indicating the smoothness of the obtained perovskite film.

**Fig. 5:**
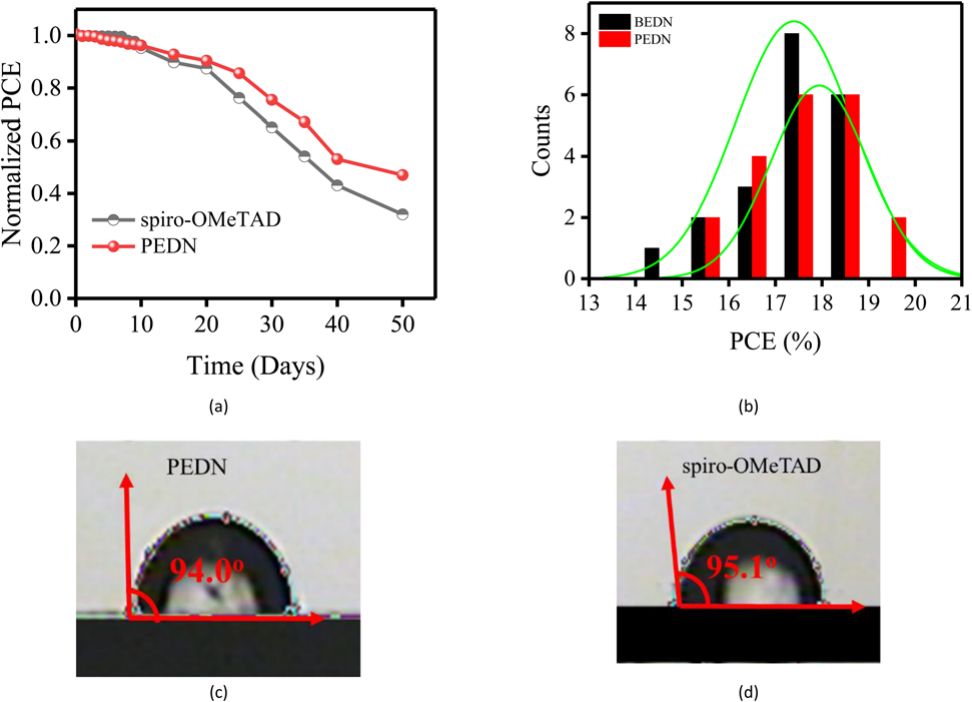
(a) The stability test of the unencapsulated PSCs by PEDN and spiro-OMeTAD. (b) Histogram of PCEs based on PEDN and BEDN measured for 20 cells. (c) and (d) Water contact angles of PEDN and spiro-OMeTAD, respectively.

The long-term stability of devices incorporating a novel HTM was assessed by exposing them to ambient air with a controlled relative humidity of 30% for a duration of 50 days. Fig. 5a demonstrates that after 10 days of exposure to ambient air, the PCEs of devices employing PEDN and spiro-OMeTAD as HTMs exhibited comparable stability, retaining approximately 95% of their initial PCEs. After 20 days, the PCEs of spiro-OMeTAD and PEDN devices had decreased to 87% and 90%, respectively. The findings demonstrate that incorporating conjugated segments into the molecular structure of HTM is a promising strategy for enhancing the stability of photovoltaic devices. Fig. 5b presents histograms depicting the distribution of PCEs obtained from 20 individual PSCs fabricated using PEDN and BEDN. The fabrication of PSCs employing PEDN and BEDN demonstrated good reproducibility, with average PCEs of 17.94% and 17.29%, respectively. Contact angle measurements between H_2_O and HTMs provide insights into the hydrophobicity of HTMs, which play a critical role in the performance and stability of PSCs. Fig. 5c and d demonstrate that the contact angle of PEDN (94°) is comparable to that of spiro-OMeTAD (95.1°), indicating its high hydrophobicity. This hydrophobicity enables PEDN to effectively prevent the penetration of moisture and oxygen, contributing to the long-term stability of PSCs under storage conditions.^39^ The lower contact angle of BEDN (83.2°) compared to PEDN can be attributed to the presence of benzyl groups in BEDN,^20^ which disrupt the planarity of the molecule.

Moreover, compared to spiro-OMeTAD, the significantly lower cost of preparing PEDN HTM is a key advantage, making it a promising candidate for the cost-effective fabrication of PSCs. As detailed in Table S1,† the estimated cost of 1 g of PEDN is $4, which is significantly lower compared to that of spiro-OMeTAD ($273.62 g^−1^).^29,40,41^ This substantial price difference can be attributed to the fewer synthetic steps required to prepare PEDN (one-step synthesis) and the absence of expensive catalysts (*e.g.*, Pd/bis(diphenylphosphino)-l,l′-binaphthyl (BINAP)) in its synthesis.

## 4 Conclusions

In summary, a novel naphthalene-based HTM, PEDN, was synthesized as a cost-effective and high-performance HTM for PSCs. The CV measurements and DFT calculations revealed that HOMO energy level of PEDN is more compatible with the HOMO energy level of the perovskite absorber than that of the commonly used HTM, spiro-OMeTAD. The PCEs of solar cells employing PEDN exhibited comparable values to those of devices utilizing spiro-OMeTAD. Notably, the PCEs of the PEDN-based solar cells were significantly higher than those of devices fabricated with BEDN as the HTM. Furthermore, the thermal stability of PEDN was found to be superior to that of both BEDN and spiro-OMeTAD. In addition to the high efficiency of PEDN-based solar cells, the cost of preparing this HTM is significantly lower than that of spiro-OMeTAD, highlighting its economic viability. The cost of a product is a crucial factor in determining its commercial feasibility.

## Conflicts of interest

There are no conflicts to declare.

## Supplementary Material

RA-014-D4RA03173C-s001
