# Supplementary material for: Improving the performance of perovskite solar cells by extending π-conjugation system
Source: RSC Adv. 2024 Jun 18;14(27):19083–9. doi: 10.1039/d4ra03173c (PMC11184581; doi:10.1039/d4ra03173c)
Supplement: RA-014-D4RA03173C-s001 [file RA-014-D4RA03173C-s001.pdf]

Electronic Supplementary Material (ESI) for RSC Advances.
This journal is © The Royal Society of Chemistry 2024

Improving the Performance of Perovskite Solar Cells by Extending Π-Conjugation System

Babak Pashaei[1,*]

[1] Department of Inorganic Chemistry, Faculty of Chemistry, University of Mazandaran, Babolsar, Iran; B.pashaei@umz.ac.ir

* Correspondence: B.pashaei@umz.ac.ir

1. Experimental

1.1 Materials

All the chemicals, reagents, and solvents were purchased from Sigma-Aldrich or Merck Companies and used without further purification.

1.2 Instrument and Measurements

^{1}H NMR and ^{13}C NMR spectra were recorded on Bruker Advance 400 MHz spectrometers with chemical shifts against tetramethylsilane (TMS). UV/Vis spectra were recorded on an Ultrospec 3100 pro spectrophotometer in $CHCl_3$ solution. AvaSpec-125 spectrophotometer was also used to measure emission spectra. Cyclic voltammetry was recorded on a SAMA500 potentiostat electrochemical analyzer with a normal three electrode system consisted of a platinum working electrode, a platinum wire counter electrode and a saturated calomel reference electrode. Tetrabutylammonium perchlorate (0.1 M in chloroform) was used as the supporting electrolyte. The current density–voltage (J–V) curves were measured using a solar simulator (Newport, Oriel Class A, 91195A) with a source meter (Keithley 2420) under 100 mA cm^{-2} illumination (AM 1.5G) and a calibrated Si-reference cell certificated by NREL.

1.3 Fabrication of Cell

Perovskite solar cells were fabricated on fluorine-doped tin oxide (FTO) coated glass substrates. Part of the glass substrate coated with FTO was etched with Zn powder and HCl 2 M ethanol solution. Then, the substrates were washed carefully with distilled water, detergent, acetone, ethanol, isopropanol. On these substrates, a solution of HCl and titanium diisopropoxide bis(acetylacetonate) in anhydrous ethanol was coated with spin-coating method at 2000 r.p.m. for 30 s. Then, the substrates were heated at 500 °C for 30 minutes and cool down to room temperature. Mesoporous TiO_2 layer diluted in ethanol was deposited by spin-coating at 2000 r.p.m. for 10 s to achieve 300-400 nm thick layer. After that, the substrates were sintered again at 500 °C for 30 minutes. The PbI_2 solution was coated on mesoporous TiO_2 layer for 5 s at 6500 r.p.m. and dried at 70 ℃. Then the CH_3NH_3I solution prepared in isopropanol was spin-coated on the previous layer for 20 s at 4000 r.p.m. and dried at 100 ℃. Following this step, HTMs were deposited by spin-coating at 4000 r.p.m. for 20 s. The HTM solutions were prepared by dissolving the HTM in chlorobenzene at a concentration of 78 mM, with the addition of 18 μL LiTFSI (from a stock solution in acetonitrile with concentration of 1.0 M), 29 μL of tert-butyl pyridine (from a stock solution in

chlorobenzene with concentration of 1.0 M). Finally, a 80 nm Au electrode was deposited by thermal evaporation under high vacuum.

1.4 Computational method details

The ground-state geometries were fully optimized using density functional theory (DFT) with the B3LYP hybrid functional at the basis set level of 6-31G*, and the frontier molecular orbitals were drawn using an isovalue of 0.03 a.u. All calculations were performed using Gaussian 09 package in the Power Leader workstation. The molecular orbitals were visualized using Gauss View 5.0.8.

Mobility Measurements

The SCLC method was applied to investigate the charge transport in the HTMs according to reported literature.[1] The devices based on HTMs were fabricated with the structure ITO/PEDOT:PSS/HTM/Au. To prepare the ITO coated glass substrates, the same method as the solar cell preparation method was used. After spin-coating the poly(3,4-ethylenedioxythiophene) polystyrene sulfonate (PEDOT:PSS) layer onto the substrates, the HTM films were deposited by spin-coating the HTM solutions in anhydrous chlorobenzene. Finally, a 80 nm Au electrode was deposited by thermal evaporation under high vacuum. The hole mobility of HTMs has been investigated according to literature. The hole mobility values were calculated using the Mott-Gurney law.

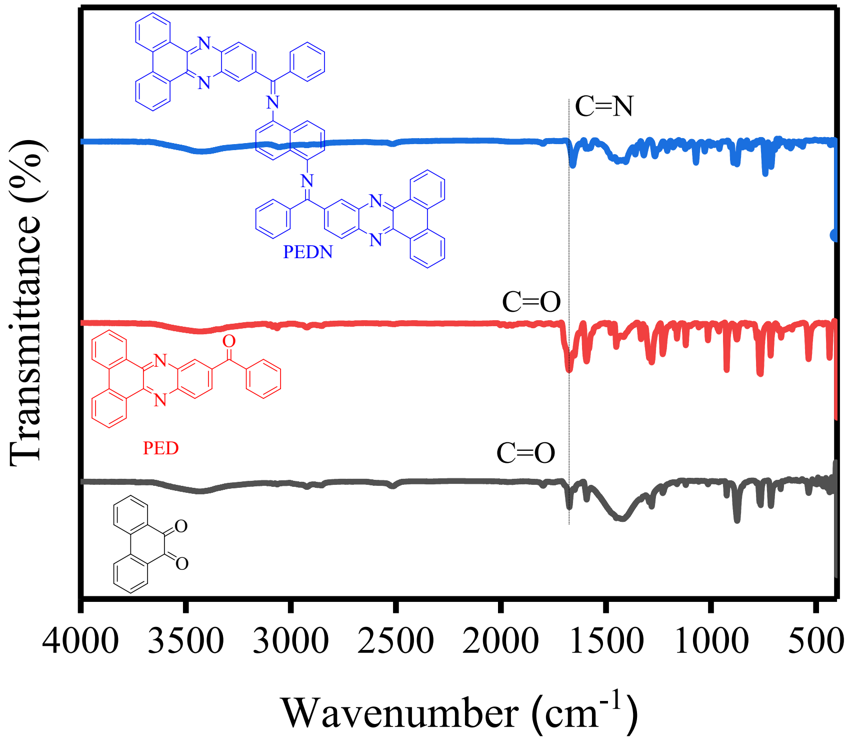

Figure S1. FTIR spectra of 9,10-phenanthrenequinone, PED, and PEDN.

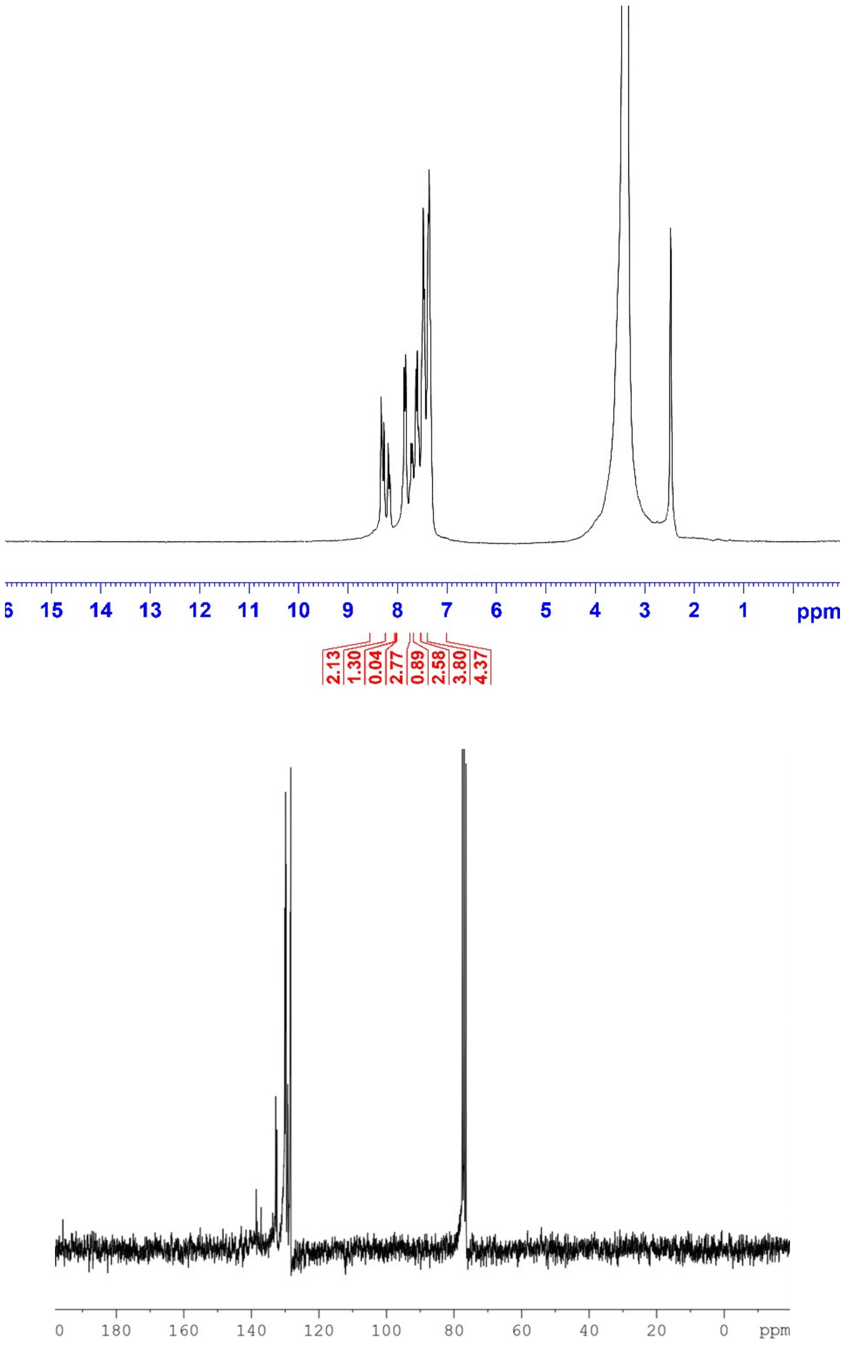

Figure S2. ^{1}H/ ^{13}C NMR of PEDN in $CDCl_3$ solvent.

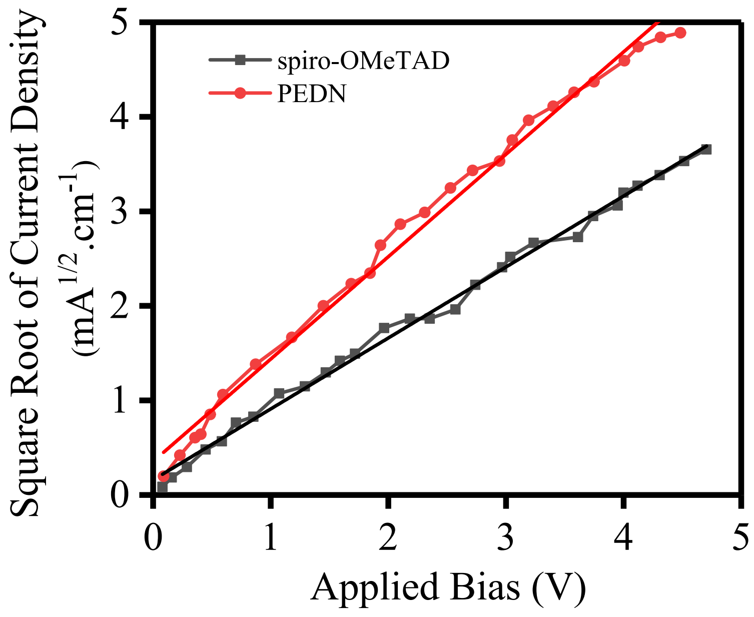

Figure S3. J $^{1/2}$ -V curves of PEDN and spiro-OMeTAD-based devices.

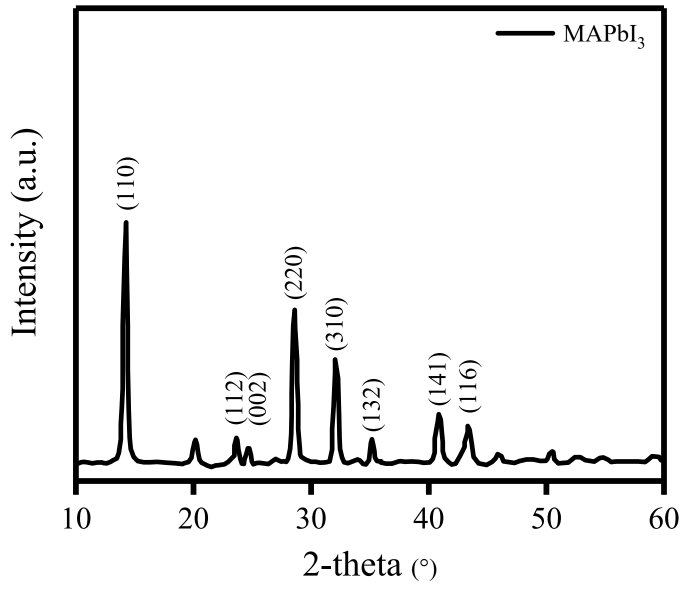

Figure S4. X-ray diffraction pattern of FTO/TiO2/MAPbI3/spiro-OMeTAD structure.

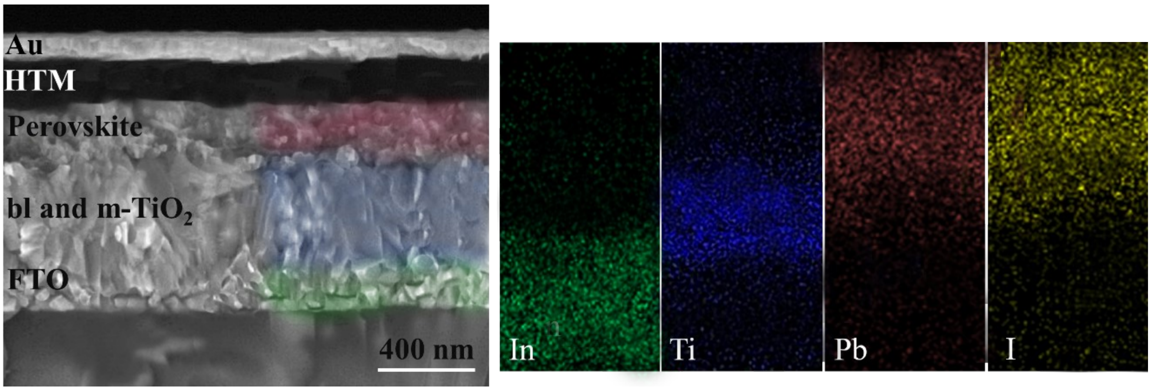

Figure S5. Cross-sectional SEM images and corresponding EDX mapping images of the solar cell, indicating the distribution of elements In, Ti, Pb, and I.

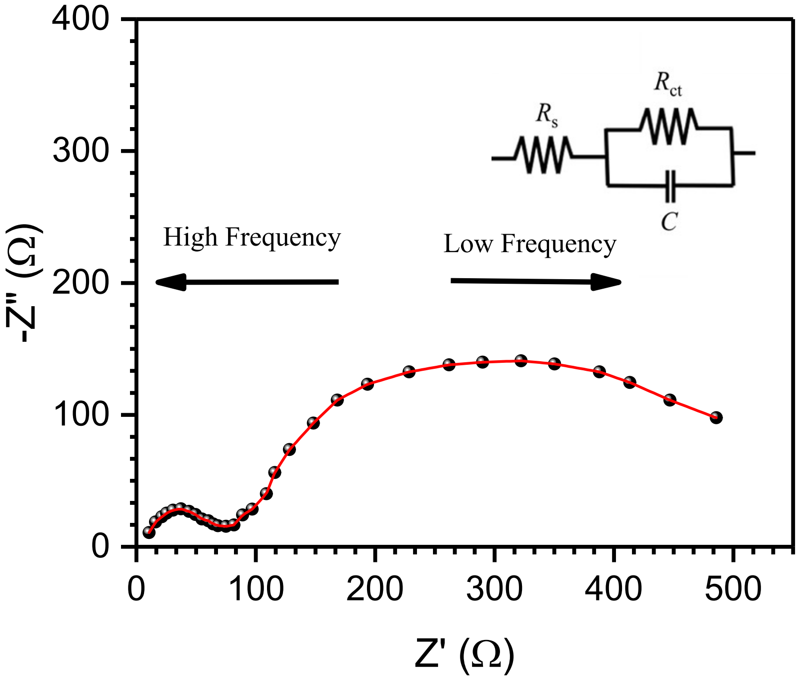

Figure S6. Nyquist plot of PSCs based on PEDN (V = 0.6 V).

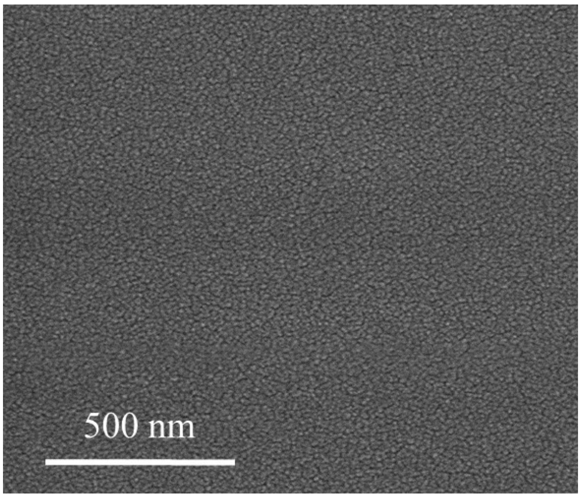

(a)

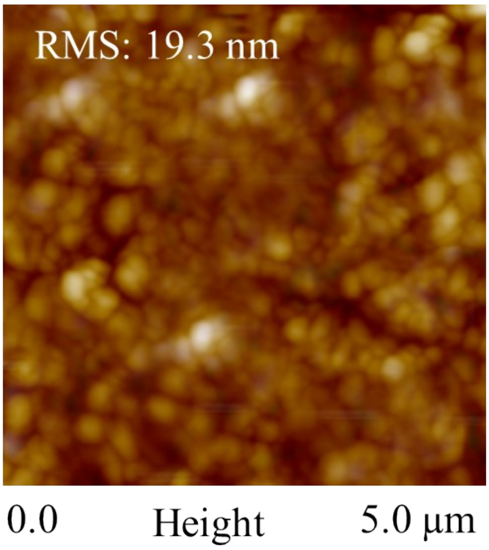

(b)

Figure S7. FE-SEM (a) and AFM (b) images of the top view of the solar cells prepared with PEDN.

Table S1. Survey of estimated chemical synthesis cost and waste streams for different HTMs.

HTM	Steps	Material Cost ($/g)	Cost per m^2 ($/$m^2$)	Chemica1 Waste (kg/g)	Commercial Price ($/g)
PEDN	2	1.80	0.4	0.45 (0.0)	-
spiro-OMeTAD	6	92	39.46	3.6 (1.0)	170-425

1. T. Leijtens, I. K. Ding, T. Giovenzana, J. T. Bloking, M. D. McGehee and A. Sellinger, *ACS Nano*, 2012, **6**, 1455-1462.
